# Animal Crossing and COVID-19: A Qualitative Study Examining How Video Games Satisfy Basic Psychological Needs During the Pandemic

**DOI:** 10.3389/fpsyg.2022.800683

**Published:** 2022-04-01

**Authors:** Andrew Z. H. Yee, Jeremy R. H. Sng

**Affiliations:** ^1^Humanities, Arts, and Social Sciences, Singapore University of Technology and Design, Singapore, Singapore; ^2^Wee Kim Wee School of Communication and Information, Nanyang Technological University, Singapore, Singapore

**Keywords:** video games, self-determination theory, psychological needs, COVID-19, mental wellbeing

## Abstract

The COVID-19 pandemic has affected the way many people live their lives. The increasing amount of time spent indoors and isolated during periods of lockdown has been accompanied by an increase in the time people spend playing video games. One such game which soared in popularity during the early stages of the pandemic was *Animal Crossing: New Horizons*. Through semi-structured interviews with players, and using a theory-informed qualitative analysis, we document and examine players’ motivations and experiences playing *Animal Crossing: New Horizons* during the pandemic. Findings suggest that playing the game helped satisfy various psychological needs—autonomy, relatedness, and competence—as described by Self-Determination Theory. Conversely, players stopped playing the game when they found that their psychological needs were thwarted or better met through other activities. Our findings offer support that video games can offer psychological relief in stressful contexts by providing opportunities for people to satisfy key psychological needs. Theoretical and practical implications are discussed.

## Introduction

A mental health epidemic occurred against the backdrop of strict movement controls enacted by various governments in efforts to tackle the COVID-19 pandemic. Containment measures targeted at reducing the rate of viral transmissions have led to greater time spent at home (Lades et al., 2020). Such measures have brought about profound changes to the way we live our lives, leading to substantial negative psychological consequences arising partially from greater social isolation and loneliness (Brooks et al., 2020; Pietrabissa and Simpson, 2020). Among the many coping behaviors individuals have adopted to deal with these changes, playing video games has—depending on who one asks—been argued to be both a panacea and a harm (Yee and Sng, 2020; De Pasquale et al., 2021; Lewis et al., 2021; Nugraha et al., 2021; Reynolds, 2021; Zhu et al., 2021).

Among critics, video games are considered to be harmful in various ways. First, some researchers suggest that the negative effects of video games—especially among children and youths—come about as it *displaces* time spent on other more productive and healthy activities such as physical activity and academic activities, among others (Gentile, 2011). Related to this is pathological gaming—also called Internet Gaming Disorder (IGD)—which refers to excessive and problematic gaming behavior (Lemmens et al., 2015). IGD may be associated with wellbeing outcomes such as satisfaction with life and loneliness (Lemmens et al., 2015). Beyond the *general* negative effects of gaming, there is also considerable debate as to the effects of violent video games on aggression. According to the General Affective Aggression Model (GAAM), extended and repeated exposure to violent video games could contribute to an aggressive personality and greater manifestations of aggressive behaviors by affecting people’s aggressive beliefs, perceptual schemata, expectation schemata, behavior scripts, and desensitizing aggression (Anderson and Dill, 2000).

Against this backdrop of research, it is important to note that the estimated number of people screened to have gaming disorder is relatively low (Stevens et al., 2021; Kim et al., 2022), with some research suggesting that pathological gaming could potentially be caused by poor psychosocial wellbeing in the first place (Lemmens et al., 2011). There is also substantial disagreement among scholars about the effects of violent video games on aggression, with several studies showing null or small effects (Kühn et al., 2019; Mathur and VanderWeele, 2019).

Turning their attention away from examining the *negative* effects of video games, some researchers have suggested that video games can facilitate positive functioning and flourishing (Jones et al., 2014). Among them, the conceptual lenses adopted tend to diverge from examining video game play in terms of time spent. Instead, scholars have examined how video games and their unique affordances enable players to experience positive emotions, autonomy, connectedness, and accomplishment (e.g., Ryan et al., 2006; Przybylski et al., 2012; Jones et al., 2014; Oliver et al., 2016; Johannes et al., 2021). For example, some scholars have examined how video games can facilitate bonding and communication between family members, potentially contributing to better mental wellbeing (Wang et al., 2018).

Regardless of whether one is convinced that video game play is psychologically harmful or beneficial, it is clear that the pandemic has seen a rise in the amount of time people spent playing them (Ellis et al., 2020; Barr and Copeland-Stewart, 2021). Video game sales have skyrocketed during the pandemic, with game companies such as Nintendo reporting more than 400% increase in profit compared to the previous year (Hackett, 2020). Play time has increased as well, with players in the United States reporting as much as a 45% increase in game time during quarantines (Clement, 2021). As a result, researchers have started examining the frequency and impact of video gameplay during COVID-19 (e.g., Lewis et al., 2021).

Despite this increased interest, most studies have adopted quantitative approaches to understand video game play during the pandemic (e.g., Kim, 2021). There is a paucity of studies utilizing in-depth qualitative approaches which provide rich and nuanced understanding of video game play motivations, players’ experiences, and its effects during the pandemic. The massive popularity of the video game *Animal Crossing: New Horizons* (*ACNH*) during the pandemic offered a unique context in which to study how video games offer a platform for dealing with pandemic-induced frustration of psychological needs. Using Self-Determination Theory as our framework (Ryan et al., 2006), we aim to provide a comprehensive account of *how* and *why* players played *ACNH* during the pandemic, and how they perceived it affected their wellbeing as they adapted to various pandemic-induced changes to their lives. Our hope is that this study enriches our understanding of the potential in which video games can serve as therapeutic platforms during social isolation.

### Animal Crossing and the Pandemic

ACNH is one of the top selling video games in the Nintendo Switch library, selling over 31 million copies over 9 and a half months (Orland, 2021). With a Pan European Game Information (PEGI) rating of 3, it is a social life simulation game where one is free to build a virtual life on their own island and do anything they wish within the parameters of the game. This includes gathering or crafting items, completing collections of bugs, fishes, or fossils in a virtual museum on their island, earning in-game currency to purchase virtual items, customizing their avatars, designing their own clothes and items, and personalizing their island. They can also visit and interact with other players through the game, exploring others’ islands, or hang out virtually. Unlike other video games, social simulation games like ACNH are open-ended with no concrete way to “win,” with players free to set their own goals within the game. Hence, “completing” the game involves meeting one’s own goals.

ACNH was released on 20th March 2020, coinciding with the early stages of the COVID-19 pandemic. It made for a unique situation which benefited the developers greatly, as measures aimed at restricting the spread of the virus led to people spending an increased amount of time at home. This led to record sales as people looked for ways to spend their time indoors (Gartenberg, 2020). Beyond that, the surge in the popularity of video games like ACNH also offered researchers an opportunity to examine the role video games play in people’s lives during the pandemic.

Over the course of the year, anecdotal evidence suggests that video games played a crucial role in helping many individuals through the pandemic. Some commentators suggested that video games were positive distractions keeping people occupied and mentally engaged (Gault, 2020; Gregory, 2020; Tassi, 2021). Despite declaring gaming disorder an addictive behavior, the World Health Organization (WHO) during the height of the pandemic also promoted playing video games as a way to encourage physical distancing (Canales, 2020). To explain the surge in popularity of video games like ACNH, some have suggested that games like ACNH helped people feel more in control amidst global uncertainty and offered platforms where people could socially connect (Lufkin, 2020; Stieg, 2020; Kelly, 2021). Stories of people across the world holding birthday parties, going on dates, holding protests, and even getting married on ACNH spoke about how video game platforms enabled intimate social interactions across physical distances (Garst, 2020; Lufkin, 2020; Liang, 2021).

### Basic Psychological Needs Theory

According to Self-Determination Theory’s Basic Psychological Needs Theory (BPNT), humans have three foundational and innate psychological needs—autonomy, relatedness, and competence (Deci and Ryan, 2000). Central to the BPNT is the empirically validated assertion that the satisfaction and frustration of these basic needs can impact individual growth, adjustment, and wellbeing (Ryan and Deci, 2017).

Briefly, the need for autonomy refers to individuals’ need to feel that their actions are volitional and resonate with one’s sense of self. The satisfaction of the need for autonomy is characterized by the experience of freedom and integration (Deci and Ryan, 2000). The need for relatedness refers to a human being’s fundamental desire to feel connected to other people - that there are other people one cares for and who cares for them. Finally, the need for competence refers to the need for individuals to feel effective by applying and extending skills.

While extant research has found that satisfying these various needs have important positive effects on wellbeing (Reis et al., 2000; Church et al., 2013), feelings of stress and ill-being can arise when environmental conditions thwart these basic needs (Weinstein and Ryan, 2011). The COVID-19 pandemic, along with government measures to control its spread, has perpetuated such negative environments for many people across the world. Nationwide lockdowns and widespread surveillance thwart people’s feelings of autonomy and freedom and significantly reduces people’s ability to connect with others, while job insecurity can frustrate individuals’ need for competence (Calvo et al., 2020; Cantarero et al., 2021; Hesse et al., 2021).

Despite the impact of the COVID-19 pandemic on wellbeing, researchers have found that fulfilling basic psychological needs, through simple interventions, can provide a useful buffer to help individuals cope with associated stresses (Cantarero et al., 2021). Indeed, others have noted that feelings of autonomy, relatedness, and competence continue to play important roles in people’s satisfaction with life (Šakan et al., 2020).

### Video Game Play and Fulfillment of Basic Psychological Needs

In direct contrast to the needs-thwarting environment of the pandemic, videogames afford players virtual environments which can satisfy their need for autonomy, relatedness, and competence (Ryan et al., 2006; Tamborini et al., 2011). Specifically, videogames such as ACNH provide a multitude of in-game player choices which support autonomy, social interaction features which support relatedness, and in-game challenges which can foster feelings of competence (Przybylski et al., 2010). The ability for videogames to offer a respite, along with the extended periods of time spent indoors or at home, meant that for some, it could be used as a source to fulfill these fundamental needs and contribute to better wellbeing.

Indeed, Johannes et al. (2021), using objective telemetry videogame play data, found that time spent playing ACNH was positively associated with affective wellbeing, which was measured *via* self-report measures assessing positive and negative feelings experienced in the previous two weeks. In another study, Barr and Copeland-Stewart (2021) conducted an online survey of video game play during the pandemic and found that respondents generally felt that playing video games had a positive impact on their wellbeing. They also found support for the proposition that feelings of control—or what they term *agency*—as well as opportunities to *socialize*, were important motivations to playing videogames during the pandemic, reinforcing the idea that videogames acted as a source for psychological need satisfaction where options were limited.

Ellis et al. (2020) noted that large proportions of players felt that playing videogames fostered better mental health during the pandemic, with it being used for emotional coping and for maintaining social connections with others. Lewis et al. (2021) specifically examined ACNH players during COVID-19 and found that social interaction through the game facilitated lower levels of loneliness and anxiety. Relatedly, researchers have also found that participating in social virtual worlds with others during the pandemic can facilitate feelings of relatedness, as well as reduce individuals’ anxiety of contracting COVID-19 (Barreda-Ángeles and Hartmann, 2022; Paul et al., 2022).

### The Current Study

As described above, several researchers have examined the positive impact videogames and virtual social worlds have on mental wellbeing during the pandemic, with some suggesting that their ability to fulfill basic psychological needs as described by BPNT is key (Johannes et al., 2021; Barreda-Ángeles and Hartmann, 2022; Paul et al., 2022).

Despite this, extant studies have largely utilized quantitative approaches to understand the effects of videogames on wellbeing. For example, Johannes et al. (2021) utilized large-scale telemetry data and the relationships between play time and different constructs relating to wellbeing. Others have looked at how the concepts of spatial and social presence related to feelings of relatedness, self-expansion, and enjoyment (Barreda-Ángeles and Hartmann, 2022). Existing studies which have attempted to account for video game experience using qualitative approaches have also tended to rely on survey responses to open-ended questions, rather than extracting in-depth information on *how* video game play specifically contributes to wellbeing (Ellis et al., 2020; Barr and Copeland-Stewart, 2021). Furthermore, scholars have argued that there is a need for researchers to pay greater attention to the specifics and subtleties of gameplay experience in relation to need satisfaction (Conway and Elphinstone, 2017).

To fill these gaps, we utilized a theory-driven qualitative approach and conducted in-depth interviews with ACNH players across the world through the lenses of BPNT. As ACNH was incredibly popular during the early stages of the pandemic, it offered a useful context for researchers to gather in-depth accounts of how it fit into the lives of players during a time when psychological needs were likely thwarted. Grounding our findings on comprehensive accounts of actual experiences and perspectives from a variety of players, we aim to understand the motivations driving players to ACNH during the pandemic, and *how* their need for autonomy, relatedness, and competence were met through these experiences.

## Materials and Methods

Throughout the study, we adopted a post-positivist paradigm (Guba and Lincoln, 1994), where the design, interview process, and data analysis were informed by an explicit theoretical framework—BPNT. Semi-structured online interviews were conducted with 17 ACNH players between the ages of 18 and 34. Participants were primarily from Singapore, with one participant each from the United States, the United Kingdom, and Taiwan. Information about the participants, along with their pseudonyms, is presented in Table 1. After obtaining ethics approval to conduct the study through our university’s Institutional Review Board (IRB-20-00321), we posted study invites to different online ACNH communities to recruit interviewees. These included the r/AnimalCrossing community on the social news aggregator and discussion site Reddit, the ACMayors server on the Discord platform, and on several ACNH communities on Facebook. Participation was open to all ACNH players, since we were interested in understanding the experiences of players from a range of different profiles, gameplay experience, and habits. During the recruitment process, we also invited users who posted on these communities to take part in the study *via* direct messaging.

**Table 1:** Information about participants.

Pseudonym	Gender	Age	Country	Living arrangements	New to gaming
Chloe	Female	25	Singapore	With People	No
Taylor	Female	34	Singapore	With People	No
Harold	Male	25	Singapore	With People	No
Yasmin	Female	20	Singapore	With People	No
Natalie	Female	29	Australia	With People	No
Daryl	Male	26	Singapore	With People	No
Victoria	Female	25	Singapore	With People	No
Jenna	Female	23	United States	With People	No
Graham	Male	29	Singapore	With People	Yes
Clarissa	Female	21	Singapore	With People	Yes
Denise	Female	25	Singapore	With People	No
Ella	Female	21	Singapore	With People	No
Hilda	Female	19	United Kingdom	With People	No
Lewis	Male	28	Taiwan	Alone	No
Thomas	Male	30	Singapore	With People	No
Vanessa	Female	26	Singapore	With People	Yes
Chris	Male	26	Singapore	With People	No

Participants’ informed consent was obtained prior to the start of the interview. During the informed consent procedure, participants were clearly briefed about the duration and the general topics that would be discussed during the interview. Most importantly, it was emphasized to participants that their decision to participate was completely voluntary and that they could reject answering any questions or stop the interview if they felt uncomfortable or distressed when discussing their experiences playing ACNH during the pandemic.

Interviews were conducted between August 2020 and July 2021 using online video or audio calls *via* videoconferencing platforms Discord and Zoom, depending on participants’ preferences. An interview guide was crafted with a focus on open-ended questions designed to elicit in-depth information from participants in relation to their ACNH gaming experience. Each interview began with asking participants what they would say if they had to write an online review for ACNH. Their response to this question determined the order of subsequent questions, which explored their experience as a ACNH player including their gameplay habits, emotional experiences, and observations about other players. The duration of each interview ranged between 20 and 50 minutes. These interviews were recorded, transcribed, and for one participant from Taiwan, translated from Chinese to English.

To analyze the data, we utilized a two-step process as described in other theory-driven qualitative studies (e.g., MacFarlane and O’Reilly-de Brún, 2012; Moran et al., 2014). First, the first author used the constant comparison approach to identify emerging themes and concepts that were open-coded (Strauss and Corbin, 1990). At this stage, he read the transcripts line-by-line and coded themes as they emerged. He did this iteratively, with codes being compared between transcripts and synthesized into higher-order categories when necessary. In the second step, he mapped the themes onto the BPNT constructs. Following the principles of the constant comparison approach, he continued to map these emergent themes to the BPNT constructs using an iterative process, constantly moving back and forth between the data, the emergent themes, and the BPNT literature. Emergent themes which did not initially map onto the core BPNT constructs were set aside for consideration and discussion, until we were confident that we were not force-fitting the data into predetermined categories. An abductive approach was adopted to handle data which did not map onto the BPNT constructs (Reichertz, 2010). First, tentative themes were proposed by the first author to group some of the coded data. This was followed by a discussion between the two authors about how well these pieces of data fit with the proposed themes in relation to the broader literature. As a result, our analyses yielded some themes outside of the core BPNT.

Throughout the process of data analysis, the second author took up the role of a critical friend, acting as an important resource for challenging and refining the interpretations made by the first author in his initial coding of the data and the mapping of the data to the BPNT constructs (Smith and McGannon, 2018). This was particularly useful to ensure that multiple plausible interpretations were considered during the analysis, as the second author was involved in many of the interviews and connected directly with the participants. In total, over 190 pages of transcripts were analyzed using *QCoder*, an *R*-based qualitative coding package which allows researchers to create codebooks, code textual data, and export them into different formats for collaboration (Waring et al., 2021).

## Findings

Five main themes emerged from the analysis. The first three themes aligned with previous research on SDT and videogame play (e.g., Przybylski et al., 2010), with the need for autonomy, relatedness, and competence expressed as key motivations for playing ACNH during the pandemic. Playing ACNH satisfied these needs through specific game experiences, from enabling a sense of freedom and escape amidst physical isolation, to facilitating human connections between friends and strangers. Next, participants sought to enhance need satisfaction through the enhancing of self-presence. Finally, participants noted that they stopped playing ACNH when their psychological needs were thwarted or better met through other activities. Table 2 provides a summary of the themes and sub-themes derived from the data, along with the exemplar quotes from the data.

**Table 2:** Key themes, sub-themes, and specific quotes.

Theme	Sub-theme	Exemplar
Satisfying the need for autonomy	Sense of escape and virtual substitutes for restricted activities	“Hm, I think it’s because during [the] lockdown, no one could travel. But in Animal Crossing… it feels so real, you go into an airport, then you take an airplane, and you fly. I kind of like that experience a lot… because it feels like you are flying, you know?”
	Freedom of expression	“In the game, I set aside a small memorial for my [stillborn] daughter, so, I guess going in from time to time does have that…I think…where you have a place where it’s not anybody else but yours.”
	Freedom to set one’s own goals	“The selling point of Animal Crossing is that I did not have to conform to any goal, I do not have to achieve a particular objective…I can just do whatever I want, set my own goals.”
Satisfying the need for relatedness	Maintaining existing connections	“…hang with people you cannot meet in-person…”
	Sense of connection to a broader community	“But there’s another aspect [to] the community, in general, with people [who] play Animal Crossing. It’s not like we *literally* play the game together, but it’s more of the…culture behind it. People celebrate the game through like internet posts on Reddit.”
	Enabling new connections	“Of course, I looked for friends online to play with. We got close and even met up in real life a few times.”
	Non-playable characters	“Okay, I know, it is just a piece of code, I know that this game has no real bearings on real life, but you know whenever I…upset a villager or the villager ask where have I been, I actually do kinda feel bad about it.”
Satisfying the need for competence	Setting and meeting goals	“I feel like I get a lot of my sense of self-worth from [in-game] achievements. In the real world that is very hard to control…but in the game, you know what those rules are.”
	Social aspects of competence	“If the island was just for me, I would not put in so much effort into designing it.”
Blurring of lines between the virtual and the real		“In the house you can do up your own wallpaper. Put your own furniture…you can even customize your own designs…I work in a preschool, so just for fun, I create my kids’ uniform inside the game.”
Needs frustration and reduced motivation to play ACNH		“[There] is a very big cancel-culture [in Animal Crossing]. It is very big. If you [have] a decently big account, I mean 5 k followers on Twitter and you make a mistake, they will actually go and mass-report the account. It is quite toxic to be honest.”

### Satisfying the Need for Autonomy

Through narratives shared by participants, we found that ACNH facilitated a sense of autonomy in three ways. Participants recounted that ACNH offered a feeling of escape and distraction from the restrictions and stresses of the pandemic and day-to-day life, provided a platform with the freedom to artistically express oneself, and enabled them to set and fulfill their own goals.

#### Sense of Escape and Virtual Substitutes for Restricted Activities

One prominent theme which emerged from participants’ recounting their experience during lockdowns was how ACNH offered virtual substitutes for pre-pandemic activities which contributed to one’s sense of freedom. Daryl—a college student—highlighted how he lived in a small room, and prior to the lockdown in his country, frequented nearby parks to decompress. He said: “So it’s just like yeah, I want to do something that is not work-related. It’s kind of like relaxing and there is no point to it. It’s like a substitute for just walking in the park.” Indeed, Victoria said that ACNH offered the experience of traveling in a time when people could not travel:

Hm, I think it's because during [the] lockdown, no one could travel. But in Animal Crossing… it feels so real, you go into an airport, then you take an airplane, and you fly. I kind of like that experience a lot… because it feels like you're flying, you know?

This sense of freedom is experienced through the spontaneity and range of possible activities in an open-world life simulator like ACNH, which is contrasted by the lack of available activities in modern cities exacerbated by the pandemic. 34-year-old Taylor said:

Um, I think for me, it really is about, um, I mean the activities we do in Animal Crossing right, so like fishing, and like swimming and like catching bugs, it's like so, plain and mundane, but yet at the same time, it's something that we don't really quite have. I mean as a working adult, everyday I'm doing a nine-to-five, I guess in many ways, maybe it helps me, I don't know, takes me away a little bit from the rat race, I think.

Indeed, previous research has highlighted how the ability of video games to offer a sense of *agency* is a key motivating factor for gaming during the pandemic (Barr and Copeland-Stewart, 2021). This was described by Graham, who said that ACNH provided him with a sense of control in a time where things were spiraling out of control. This was also expressed by Daryl, who said:

It’s almost like this quaint village, I mean it’s virtual, [but where] you can go to and *exist*.

#### Freedom of Expression

Feelings of autonomy were not only fostered through virtual substitutes of physical activities in the ACNH game world. The game mechanics, which enable a high degree of customizability, offered players a platform where they can experience substantial autonomy to experiment and express themselves. This involved not only the customization of in-game avatars, but also in the design of their entire island, as players could customize—through an in-game design tool—unique patterns for clothes, wallpapers, and road pavements, among others. This manifested not just in appearances, but also in the expression of experiences and opinions as wide-ranging as politics and personal grief. Such self-expression and ability to make choices which align with a players’ sense of self can help players experience a sense of autonomy (Przybylski et al., 2012; Conway and Elphinstone, 2019). Graham recounted how he participated in an LGBTQIA rally on ACNH through such customization tools:

My friends and I will go to Pink Dot every year… because of the pandemic, [we] obviously [couldn’t attend] a physical Pink Dot rally. I think there were a couple of LGBT activists, they designed the Pink Dot shirt. You know on Animal Crossing you can download people’s designs and templates. Because we couldn’t go to the gathering this year, what I did was that I went to download that shirt [and] wore it. Then, I went to buy like pink sunglasses, pink shoes, pink shorts. Everything. And there was this ice-cream cone lamp that was rainbow colored. I took all these LGBT-related items, put it in one spot, took a nice photo, and posted it on Instagram on the day of Pink Dot. That is my way of participating.

Taylor discussed how she recently lost a child at birth, and the customization options allowed her to express herself and grieve through the setting up of a virtual memorial on the Island.

In the game, I set aside a small memorial for my [stillborn] daughter, so, I guess going in from time to time does have that…I think…where you have a place where it's not anybody else’s but yours.

Such psychological benefits of creative self-expression are sometimes discussed in research on writing- and art-based practices for mental health recovery (e.g., Pennebaker, 1997; Stuckey and Nobel, 2010), and these experiences highlight how affordances within ACNH enabled some participants to fulfill their need for autonomy through self-expression.

#### Freedom to Set One’s Own Goals

In the discussions, participants also highlighted that ACNH differed from other games as it allowed players to determine their own purposes for playing the game, rather than meet pre-defined parameters for “winning” the game. For example, Chloe mentioned that the ability to decide what she wanted to do, such as in designing new patterns for use in in-game products, was highly enjoyable. Graham also added:

So, I have never considered myself a gamer. The selling point of Animal Crossing is that I didn’t have to conform to any goal, I don’t have to achieve a particular objective…I can just do whatever I want, set my own goals.

Other participants recounted how they enjoyed starting projects, setting their own goals, and taking their time to enjoy the process of working on these projects, which ranged from designing customized road tiles to collecting specimens for the in-game museum. As suggested by previous research, the process of setting goals for oneself can foster feelings of autonomy and intrinsic motivation (Seo et al., 2018).

### Satisfying the Need for Relatedness

One theme which emerged consistently among multiple participants was that ACNH provided a platform for human connection, which can help satisfy the basic need for relatedness. This was experienced by playing with existing connections such as friends and family, participating in online ACNH communities, connecting with strangers who also played ACNH, and developing parasocial relationships with in-game non-playable characters.

#### Maintaining Existing Connections

ACNH features both online and local multiplayer game modes. Regarding its online mode, the ability for players to virtually be with friends and family one cannot meet with due to the pandemic-related restrictions were constantly highlighted by participants. As one participant, Natalie, said, ACNH allowed her to “hang with people you cannot see in-person.”

This experience is further mediated and enhanced by game design choices. For example, the process of visiting someone else’s island involved virtually going to the airport, watching an animation of one’s seaplane flying over the island, and arriving at a destination airport, with your host likely waiting to greet you on arrival. These design choices evoked a high level of self-presence, which refers to one’s perception that they are *inside* a virtual environment. Relatedly, interacting with another person’s avatar on these virtual islands generated a sense of *social presence*, which refers to the psychological experience of virtual avatars as actual people (Lee, 2004). Chloe recounted her experience playing with friends:

I prefer to, when I'm decorating, I like to have my friends around. 'Cause I want to have…I just want to have like people's opinion, you know?

Social presence can also be muted with little to no verbal or written communication, but foster feelings of connectedness with someone else, just by being in a shared virtual space. Taylor, who was going through the death of her daughter, said:

I guess people in general don't really quite know how to deal with death for such a young person. So I guess Animal Crossing in some ways allowed me to have that platform where I can interact with people who want to interact with me but I…but it allows a certain level of distance, comfort and distractions I think…for example, if my friend, my colleague, for example, who visits me in an island right, we will, in the game, go fishing and do, I mean, we will accompany each other, or she would accompany me, but then, it wouldn't be like, having to…sigh…deal with the death or the topic of death so straightforwardly? I don't know if you know what I mean…I guess in many ways, [the] platform allowed that safe middle ground or that safe space where people can visit and mingle…and I guess in a way, try to ensure that I'm okay, but not have to broach the topic [or] the issue…

In addition to online multiplayer, players who played with friends, partners, or family members on the same device shared the same virtual island and often used it as a common activity for bonding and building meaningful connections. Taylor said that playing locally with her 5-year-old child allowed her to “enter his mind…[and] understand what he’s thinking about, what he enjoys, what he does not.” Another participant, Clarissa, mentioned how she shared different roles to her sister, such that her sister oversaw the design the island, while she tried to earn in-game currency through different tasks to fund her sister’s work. Such co-playing of videogames have been found to be related to family closeness and satisfaction, as well as increased feelings of relatedness in players (Tamborini et al., 2011; Wang et al., 2018). Beyond this, players also report reconnecting with others due to this shared interest in the game. Clarissa added:

I have a friend that I haven’t been talking to since I was in Junior College, [from about] two to three years ago. Then…he saw me posting the fossils thing on the group… he came to PM (private message) me. It was quite cute. Suddenly, [I rekindled a] friendship. Quite cool.

#### Sense of Connection to a Broader Community

One participant, Jenna, mentioned how she sometimes looked for inspiration and posted images of her island to online ACNH communities on different social media platforms. Several participants also mentioned this broader ACNH community, and their participation ranged from being passive to highly active. Passive participation involved scrolling through these communities to observe and browse other people’s island either for entertainment or for design ideas, while active participation involved posting and engaging with others in this online community. As the need for relatedness refers to an individual’s desire to feel connected to others and the broader social world, participation in such communities may help facilitate the satisfaction of such needs through the cultivation of a shared social identity and a sense of belonging (Tajfel and Turner, 1979; Jia et al., 2021).

This is also achieved through a set of common values shared by members of the community, culminating in various implicit and explicit social norms. For example, Chloe highlighted that when visiting other islands, it was inappropriate to visit in a wetsuit, as the wetsuit would enable visitors to bypass the fencing put in place to prevent them from entering more private places on someone’s island. Other norms expressed by many of the participants include how it was considered rude to trample on someone’s flowers or take stuff without asking. The emergence of such norms indicates a strong sense of community, which contributes to the satisfaction of the need for relatedness, as players may feel like they are part of something meaningful. Furthermore, when individuals contribute or maintain community norms, they may experience a sense of influence in the community (Blanchard and Markus, 2004). This sense of community was best expressed by Harold:

But there's another aspect [to] the community, in general, with people [who] play Animal Crossing. It's not like we *literally* play the game together, but it's more of the…culture behind it. People celebrate the game through like internet posts on Reddit. [That] in itself, is sort of experiencing the game, outside of the game itself, you know? It's like, when I see internet posts of people like, saying this particular villager is annoying, or they like this villager, or like, they made this custom dress from this other game and like, this is all outside the game that I'm participating in… like it's technically *off the game*. It's just media surrounding the game, but not the game itself.

#### Enabling New Connections

Participating in these communities enabled players to connect with other players. In the process of participating in online communities, players may open their islands to visitors either just for fun, or for transactional purposes, such as in trading items. However, these interactions have the potential to progress into deeper and more meaningful relationships. One participant, Ella, mentioned:

A lot of them are just Twitter friends. They have nice content and when you reply to their content, they interact with you. If you all can sort of, click in a way, you progress to conversing.

These off-game conversations have the potential to gradually become, as players put it, in-real-life (IRL) friendships, as explained by Lewis:

I used to play games with my friends from real life. However, most of my friends don’t play Animal Crossing. Of course, I looked for friends online to play with. We got close and even met up in real life a few times.

#### Non-playable Characters

While previous SDT research has examined how the need for relatedness can be met through multiplayer games with other players, few studies have examined how NPCs can help satisfy those needs (Conway and Elphinstone, 2017; Tyack and Wyeth, 2017). An emergent theme in our interviews was that participants do indeed form parasocial relationships with NPCs, which could contribute to satisfying their need for relatedness. Yasmin, 20, explained:

Okay, I know, it is just a piece of code, I know that this game has no real bearings on real life, but you know whenever I…upset a villager or [when a] villager asks where have I been [after I haven’t been playing for a while], I actually do kinda feel bad about it.

This was not a surprise, as previous scholars have argued that parasocial relationships—where a media user responds to a fictional or media figure as in a typical and real relationship—can develop between individuals and fictional characters (Horton and Wohl, 1956; Giles, 2002). Similarly, as Lee (2004) argued, feelings of social presence can extend to artificial social actors such as the ACNH NPCs. These may manifest in real emotional responses, and bear psychological effects in terms of relatedness (Conway and Elphinstone, 2017; Tyack and Wyeth, 2017). One participant, Vanessa, described this psychological impact:

I think like..ok..because different animals have different personalities…there are some animals who are really like…*ok wait…I shouldn’t call them animals*. There are some villagers [who] are like more wholesome. Like they will say very nice things, and then you will feel nice about yourself. You also get villagers who will like bring you gifts, which is so like…which is so sweet like they would…they can either mail you gifts or like they run up to you to say hi…that sort of thing. And like it makes me feel really happy and it also makes me feel like very umm…part of something. I guess?

### Satisfying the Need for Competence

While relatedness and autonomy satisfaction emerged from the discussions, some participants also noted that even though there was no way to “win” ACNH, the setting and meeting of personal goals within the game could provide feelings of competence. Furthermore, participants noted that this sense of competence can be enhanced when interacting with a broader community of players.

#### Setting and Meeting Goals

The personal goals set by participants ranged from modest ones such as completing a museum collection to highly ambitious ones such as completing every single achievement available within the game. These also varied in their contents (Vansteenkiste et al., 2006), from more extrinsic goal contents like getting a five star rating for your island from the in-game rating system to more intrinsic ones such as recreating a Japanese Zen garden to relive one’s real-world travel experience. Regardless of the content, goals set within ACNH can provide players with a sense of competence and self-worth. Natalie recounted:

Maybe it sounds a bit morbid, but I feel like I get a lot of my sense of self-worth from [in-game] achievements. In the real world, that is very hard to control…but in the game, you know what those rules are, so you know that in a grind situation, something very long term like [Animal Crossing], if I for example, have to catch 5,000 fish, then I know if I just keep at it over several days or probably months, if I do that, I will get that little sort of…you’ve done it at the end…and I’ll know I’ll be able to check that little line of the list…and I really like that feeling.

#### Social Aspects of Competence

One consistent finding was that the need for competence often involved a social dimension. Participants described a sense of pride in their islands and noted that working on their goals were particularly motivating when it meant that there would be visitors to their island. Lewis discussed his project of creating mazes in the game for visitors to experience, and his sense of achievement when others work on them. Ella said: “If the island was just for me, I would not put in so much effort into designing it.” Echoing this sentiment, Chloe said:

I have this very small spot on the island that is like [a] Japanese Zen Garden area…That is something I am proud of…like if I were to invite people to my island now, I will restrict them to that spot. Because it is nice. That is something that I am proud of.

### Blurring of Lines Between the Virtual and the Real

Beyond the satisfaction of basic psychological needs, participants discussed how the lines between what is virtual and what is real were sometimes intentionally blurred through player choices. Participants often incorporated aspects of their identity into the game, such as through the recreation of memories and experiences through customization tools. One participant, Denise, mentioned:

In the house you can do up your own wallpaper. Put your own furniture…you can even customize your own designs…I work in a preschool, so just for fun, I create my kids’ uniform inside the game.

Chloe said that she designed parts of her island based on her memories traveling with family and friends:

By the time my island hit five stars, the entire island was all thematically designed to just allow me to remember like all the travelling that I've ever done, like, all of holidays with my fam, or like, holidays with my friends and all of that…if anyone were to visit my island, and I could take them on to a tour, I would just spend like the entire time… [to] explain like, okay this part I was…inspired by like…the lavender fields from Hokkaido, the Zen garden was when I visited Japan… And then…there [were] some parts that were very inspired by Taiwan…some parts that were inspired by Paris when I went there for school. And yah. So it was like, the entire thing was all very meticulously planned out…

These reflect players’ actions to experience some level of *extended self-presence* (Ratan, 2013). According to Ratan (2013), the experience of integrating one’s physical self with a virtual self-representation is described as *self-presence*. Meanwhile, *extended self-presence*—a dimension of *self-presence*—involves the process of extending some aspects of *personal identity* into a virtual representation of one’s self. Travel memories, as well as players’ occupations, reflect identity-relevant aspects of one’s self that may help player’s experience greater self-presence. Choosing to customize aspects of one’s game to include such identity-relevant concepts could increase the relevance and emotional connection one has with their virtual self-representations (e.g., Ratan and Dawson, 2016), potentially augmenting the experience of need satisfaction through ACNH.

### Needs Frustration and Reduced Motivation to Play ACNH

Consistent with BPNT, participants revealed that their motivation to play the game would drop when they encountered needs-frustrating experiences (Przybylski et al., 2010; Kosa and Uysal, 2021). This may manifest when the feeling of agency gets threatened instead of being reaffirmed. Yasmin noted that after months of playing the game daily, she felt that the daily tasks of watering the flowers, plucking fruits, and looking for certain items became a chore, since she *must* do it to reach some of her goals. Even though these goals were self-set, the daily tasks became something that was not volitional, frustrating one’s need for autonomy. She stopped playing the game soon after.

Relatedly, when participants feel that they no longer can find a sense of connection—whether with the broader community or with friends—through the game, the motivation to play will be diminished. Ella explained how she slowly stopped playing ACNH:

[There] is a very big cancel-culture [in Animal Crossing]. It is very big. If you [have] a decently big account, I mean… [about] 5,000 followers on Twitter… and you make a mistake, they will actually go and mass-report the account. It is quite toxic to be honest… The friends I made also stopped being as active…[in] the game. We do talk from time to time. Like it is not really about the game. They progressed to be more online pen-pals of sorts.

Interestingly, when the social connections made *via* ACNH ceased to exist on the platform, they shifted their interactions to other communication mediums. Some scholars have found that friendship ties enabled and formed *via* online games often go through a series of stages, with relationship building taking place on communication mediums outside of the game itself, since it facilitated more personal interactions (Lai and Fung, 2020).

Finally, some participants mentioned that they stopped playing the game when they felt that they could not achieve the goals they set for themselves, frustrating their need for competence. Yasmin said: “Well, at the start it was quite alright, though I knew I was working toward a goal. But after a while, I realized that my island looked a bit janky. And it was *really* difficult to fix so that’s where my motivation started to drop.”

## Discussion and Conclusion

Video games have the capacity to satisfy basic psychological needs, especially during times when many of these needs may be thwarted (Przybylski et al., 2010). Following the idea that video games may serve as a way for people to cope with the impacts of the pandemic, extant research have provided some evidence for the positive impact of video games during the COVID-19 pandemic (Ellis et al., 2020; Barr and Copeland-Stewart, 2021; Lewis et al., 2021). Our study extends the findings from these studies by providing a qualitatively richer account for *how* the process of need satisfaction takes place through the ACNH game.

Through the narratives of players who had been involved in the game, we found that the ACNH’s game design and mechanics enabled players to perform virtual activities which enhanced a sense of agency during a time where feelings of autonomy had been stifled due to pandemic control measures. The substitute virtual activities, customization options, and self-setting of goals allowed participants a feeling of choice and volition. This corroborates previous research which had found that in-game player choices can help support people’s need for autonomy (Przybylski et al., 2010).

Furthermore, ACNH provided a platform for people to *connect*. Players’ sense of relatedness was not only derived from playing with known contacts such as friends and family, but also from passively or actively participating in a larger community—reinforced through social norms—connecting with new friends made through the game, and even players’ parasocial interactions with NPCs. While previous studies have highlighted how video games helped players maintain social connections with others and enabled social interactions during the pandemic (e.g., Ellis et al., 2020; Barr and Copeland-Stewart, 2021), the findings from our study deepened this understanding by illustrating the breadth of possibilities in which video games like ACNH can foster psychological connectedness.

Even in a videogame with no explicit long-term objectives, ACNH could satisfy feelings of competence through goals which players set for themselves. The source of such feelings of competence may be social in nature, and come from the broader community, through the sharing of their “work.” This is unlike other videogames with explicit goals which can satisfy players’ need for competence through the game itself (Przybylski et al., 2010).

In their narratives, participants also shared how they intentionally tried to increase their feelings of *extended self-presence* by integrating facets of their real lives into the game. Such actions could possibly enhance the emotional connection between their in-game avatars and their physical selves (Ratan and Dawson, 2016), thereby augmenting their experience of need satisfaction that is embodied by their in-game characters.

Finally, while the motivation to play ACNH was facilitated by need frustration driven by the pandemic environment, players stopped playing when they felt that the platform no longer supported some of these needs. This is no surprise, since enjoyment would likely wane when feelings of autonomy, relatedness, and competence are not fulfilled (Tamborini et al., 2011). Despite this, some connections formed through the platform continued even after players stopped playing the game.

There are several theoretical and practical implications from our study, which are bound by some inherent limitations of our design and approach. Theoretically, there are few in-depth and thick descriptions (Geertz, 1973) of how videogames were used during the pandemic as a source of wellness. Our study offers an intricate and detailed look into the use of videogames for psychological wellbeing through the lens of BPNT. This may help inform future research on video games and BPNT. In our study, we found that the satisfaction of relatedness may arise not only through playing with other people, but also from the feeling of being a part of the larger ACNH community and through interactions with NPCs. Existing measures of need satisfaction through video games, such as the Player Experience of Need Satisfaction (PENS) scale, tend to only operationalize relatedness satisfaction through interactions with other players directly within the game (Ryan et al., 2006; Johnson et al., 2018). We argue that measures of need satisfaction from video games, such as the PENS, ought to include these other sources which can contribute to relatedness satisfaction.

Practically, our study extended the research on and provided more support for the potential for videogames to support essential psychological needs of humans, especially in contexts where such needs may be thwarted. As these needs are fundamental to individual wellbeing (Ryan and Deci, 2017), there is a case for exploring the use of videogames to become a source for the fulfillment of such needs when individuals may not be able to fulfill them in other ways. Needs which may be fulfilled simply from a walk in the park can be denied to people based on their circumstance, as one of our participants has experienced during a lockdown in his country.

Beyond the pandemic, situations and contexts which thwart such basic needs can result in psychological distress, and this includes patients having to undergo long-term bedrest, grieving individuals, people with mobility issues, or even incarcerated prisoners, among others (Maloni et al., 2002; Bedaso et al., 2020). Video games may provide psychological relief for individuals going through difficult emotional experiences in life. For example, ACNH with its plethora of customization options and freedom to create provided a platform which allowed Taylor a space for creative expression of her grief. This is reminiscent of art-based therapy to reduce adverse psychological outcomes (Stuckey and Nobel, 2010).

Hence, BPNT can offer a useful framework for the design of video game-based interventions to promote psychological wellbeing in these contexts (e.g., Woodall et al., 2014; Woods et al., 2017). Specifically, game designers hoping to create games which are targeted at helping people cope with difficult life experiences can include game elements which foster feelings of autonomy, competence, and relatedness. Beyond implications for game design, our study suggests that policymakers and healthcare professionals can consider the use of video games to promote mental wellbeing and use the BPNT as a framework to guide their selection of games for people who can benefit from it. The use of games can be accompanied by the development of communities where players can interact *outside* of the game to fully harness the power of games to develop a sense of belonging to a larger community, fostering feelings of relatedness.

While our study focused on need satisfaction and the possible positive effects of video game play on players, we also need to be cautious about the possible risks associated with excessive play and risky online behaviors (Lemmens et al., 2015; Gámez-Guadix et al., 2016). For example, Lewis discussed meeting people face-to-face after having known them online, while Ella spoke about the “cancel culture” that sometimes manifest in the ACNH community. Players, especially those who are younger, need to be educated about the risks of participating in online communities, including but not limited to managing cyberbullying, verbal aggression, and meeting strangers IRL (Gámez-Guadix et al., 2016; Sng, 2020). Video game players should also be aware of and distinguish between healthy gaming and pathological gaming (such as displacement, withdrawal, and conflict), so that they may be empowered to manage their gaming to reap the benefits without developing an unhealthy dependence on it (Lemmens et al., 2015).

The COVID-19 pandemic has certainly made the positive utility of video games apparent, as seen in the abrupt shift in the stance of the WHO regarding the use of video games. In the early months of the pandemic in 2020, WHO started encouraging people to stay at home and play video games (Canales, 2020). This was contrary to its previous stance in believing that video games can lead to mental health problems and cautioning people about the excessive use of video games (World Health Organization, 2020). Evidently, video games should not be said to be inherently good or bad, but the outcomes of use depend on the context and manner in which they are used. Games such as ACNH can be used in positive ways to fulfill psychological needs, especially when going through difficult emotional experiences.

## Data Availability Statement

The raw data supporting the conclusions of this article will be made available by the authors, without undue reservation.

## Ethics Statement

The studies involving human participants were reviewed and approved by Singapore University of Technology and Design. Full verbal informed consent was acquired prior to the interviews. Written informed consent for participation was not required for this study in accordance with the national legislation and the institutional requirements.

## Author Contributions

AY and JS both co-conceptualized the study, contributed to the design of the interview guide, recruited participants, interviewed participants, and contributed to the drafting of the manuscript. All authors contributed to the article and approved the submitted version.

## Funding

The open access publication fees are supported by the Singapore University of Technology and Design’s Kickstarter Initiative. No other funding was received for the research being submitted.

## Conflict of Interest

The authors declare that the research was conducted in the absence of any commercial or financial relationships that could be construed as a potential conflict of interest.

## Publisher’s Note

All claims expressed in this article are solely those of the authors and do not necessarily represent those of their affiliated organizations, or those of the publisher, the editors and the reviewers. Any product that may be evaluated in this article, or claim that may be made by its manufacturer, is not guaranteed or endorsed by the publisher.
